# Evaluation of Factors Affecting Fluoride Release from Compomer Restorative Materials: A Systematic Review

**DOI:** 10.3390/ma18071627

**Published:** 2025-04-02

**Authors:** Monika Oleniacz-Trawińska, Agnieszka Kotela, Julia Kensy, Sylwia Kiryk, Wojciech Dobrzyński, Jan Kiryk, Hanna Gerber, Magdalena Fast, Jacek Matys, Maciej Dobrzyński

**Affiliations:** 1 Medical Center of Innovation LLC, Wroclaw Medical University, Krakowska 26, 50-425 Wroclaw, Poland; kotela.agnieszka@gmail.com; 2 Faculty of Dentistry, Wroclaw Medical University, 50-425 Wroclaw, Poland; julia.kensy@student.umw.edu.pl; 3 Department of Pediatric Dentistry and Preclinical Dentistry, Wroclaw Medical University, Krakowska 26, 50-425 Wroclaw, Poland; s.roguzinska@gmail.com (S.K.); maciej.dobrzynski@umw.edu.pl (M.D.); 4 Department of Dentofacial Orthopedics and Orthodontics, Division of Facial Abnormalities, Wroclaw Medical University, Krakowska 26, 50-425 Wroclaw, Poland; wojt.dobrzynski@wp.pl; 5 Department of Dental Surgery, Wroclaw Medical University, 50-425 Wroclaw, Poland; jan.kiryk@umw.edu.pl; 6 Department of Maxillofacial Surgery, Wrocław Medical University, 50-556 Wrocław, Poland; hanna.gerber@umw.edu.pl; 7 Department of Drug Form Technology, Wroclaw Medical University, Borowska 211 A, 50-556 Wroclaw, Poland; magdalena.fast@umw.edu.pl

**Keywords:** fluoride release, compomer, dental materials, tooth

## Abstract

Objective: This systematic review evaluates the evidence on factors affecting fluoride release from compomer restorative materials to provide clinicians with insights for optimizing their use in caries prevention. Methods: In February 2025, an extensive digital search was conducted across reputable databases such as PubMed, Web of Science, and Scopus. The search utilized carefully chosen keywords: “fluoride release” AND “compomer” and followed the PRISMA guidelines. Initially, 287 articles were identified, but after applying the inclusion criteria, 34 studies were selected for review. Results: This review found that fluoride release from compomers follows an initial burst phase before stabilizing at lower levels. Fifteen studies proved that compomers release less fluoride than glass ionomer cements but more than composite resins, as concluded from six studies. The release rate is significantly influenced by pH, with acidic conditions enhancing fluoride diffusion. Some studies also highlighted the potential for fluoride recharge through external applications such as toothpaste or varnish. Conclusion: Compomer restorative materials offer a steady, moderate fluoride release that supports caries prevention. Their effectiveness is enhanced in acidic environments, supporting their use in high-risk patients.

## 1. Introduction

Research into dental materials that meet the requirements for marginal sealing, fluoride ion release to prevent caries [1], and biocompatibility ensuring compatibility with oral cavity fluids such as saliva and gingival crevicular fluid has been ongoing for many years. Additionally, these materials should possess antibacterial properties [2], appropriate hardness, and aesthetic appeal. The development of compomers was an attempt to combine the beneficial properties of glass ionomers with composite technology [3]. These materials are polyacid-modified composite resins and are classified as composite materials because they consist of ion-leachable glass (usually calcium–aluminum–fluorosilicate glass) embedded in a polymer matrix [4,5]. However, as these materials do not bond without light activation, they are not classified as glass ionomer cements [6]. Compomers are a popular restorative material used in both primary and permanent anterior and posterior teeth [6,7,8]. They are commonly applied in direct restorations as aesthetic materials, in colored restorative materials [9,10] and as orthodontic cements [11,12].

For many years, clinical studies have demonstrated the importance of fluoride in preventing dental caries. Dental caries is the most prevalent infectious disease worldwide, affecting an estimated 60% to 90% of the global population, particularly children. Due to its extremely high prevalence and substantial adverse impact on overall health, well-being, and quality of life, it is recognized as a global public health problem [13,14]. Fluoride inhibits enamel demineralization in the presence of acids produced by cariogenic bacteria and supports the remineralization of teeth [15]. The use of fluoride-releasing materials for cavity restorations and build-ups has increased significantly in recent years [1,16]. Fluoride exerts its anti-caries effect through multiple mechanisms, including enhancing remineralization, interfering with pellicle and plaque formation, inhibiting the growth of microorganisms, and reducing demineralization [1,15,16,17]. Additionally, it slows the progression of caries in the dentin toward the pulp.

**Figure 1:**
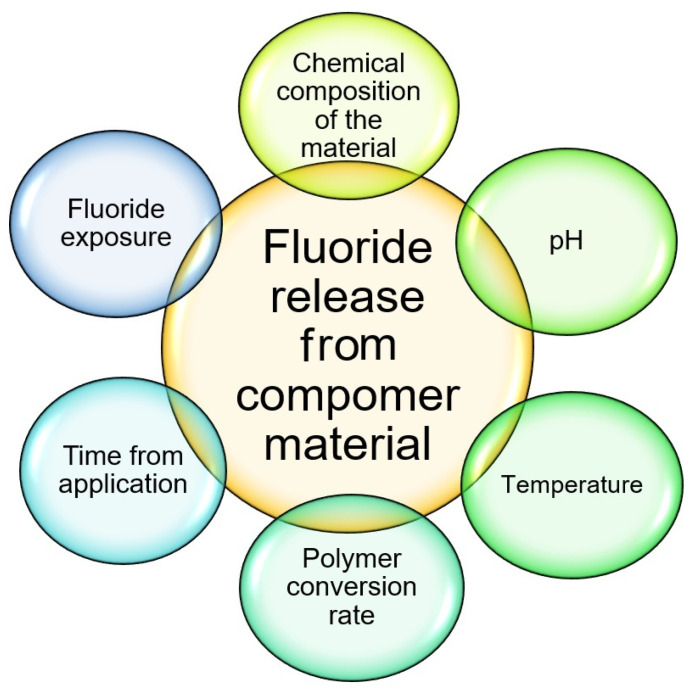
Factors affecting fluoride release.

The effectiveness of fluoride ion release in different restorative materials is highly dependent on several variables, including temperature, pH, material preparation methodology, and the type of formulation used [18] (see Figure 1). The pH environment is particularly important clinically, as it varies significantly within the oral cavity [19,20,21]. Clinical studies have shown that pH can drop to a critical level of 4.0–4.5 after glucose metabolism. The threshold for caries development is set at pH 5.5, with values between 5.5 and 6.0 considered potentially cariogenic, while pH levels above 6.0 are regarded as clinically stable for preserving tooth structure [22,23,24]. Different fluoride formulations significantly impact remineralization potential and clinical efficacy [25]. Cement-based materials provide long-lasting fluoride release, creating a sustained protective environment [20,22,26], while varnishes serve as concentrated, temporary fluoride reservoirs with enhanced adhesion to tooth surfaces [27,28]. Gel formulations offer superior penetration into porous enamel structures but require precise application protocols [29,30]. In contrast, liquid formulations, characterized by their immediate ion availability, facilitate rapid remineralization but require more frequent applications due to their shorter duration of action [27,31].

Understanding the complex factors influencing fluoride release from compomer restorative materials remains a critical challenge in restorative dentistry. While numerous individual studies have examined specific aspects of fluoride release, previous systematic reviews have primarily focused on glass ionomer cement or fluoride-releasing composites, with limited emphasis on compomers. To our knowledge, no prior systematic review has comprehensively assessed the factors influencing fluoride release from compomer materials. This systematic review aims to bridge this gap by evaluating the available evidence on fluoride release from compomers, considering key variables such as storage conditions, pH environment, and material composition. This review provides a more detailed and structured assessment compared to previous studies by systematically analyzing the trends and influencing factors. The findings offer clinicians evidence-based insights to optimize the selection and use of compomer restorations for caries prevention in clinical practice.

## 2. Materials and Methods

### 2.1. Focused Question

The systematic review followed the PICO framework as presented in [32], posing the following focused question: In case of compomer restorative materials (population) what are the factors (investigated condition) increasing the fluoride release (outcome) from these materials compared to other restorative materials (comparison condition)?

### 2.2. Protocol

**Figure 2:**
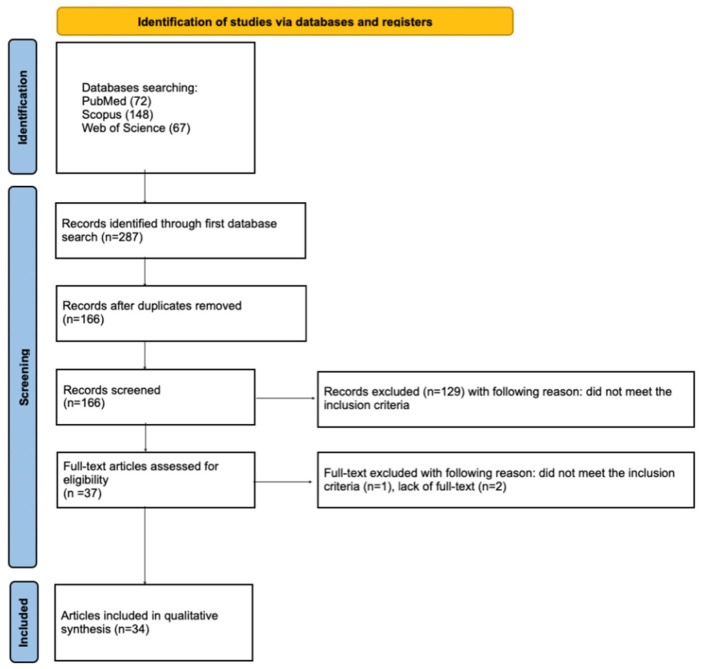
PRISMA 2020 flow diagram.

The article selection process for the systematic review was systematically detailed using the PRISMA flow diagram [33] (Figure 2). The systematic review was registered on the Open Science Framework under the following link: https://osf.io/9q4am (accessed on 20 March 2025).

### 2.3. Eligibility Criteria

The researchers decided to only include articles that fulfilled the following criteria [33]:

- Compomer restorative materials;
- Fluoride release evaluation;
- In vitro studies;
- Studies in English;
- Full-text articles.

The reviewers established the following exclusion criteria [33]:

- Not a compomer restorative material;
- Evaluation of other physical or chemical properties than fluoride release;
- Studies without a control group;
- Non-English papers;
- Systematic review articles;
- Review articles;
- Full text not accessible;
- Duplicated publications.

### 2.4. Information Sources, Search Strategy, and Study Selection

In February 2025, a systematic search was performed in the PubMed, Scopus, and Web of Science (WoS) databases to identify articles that met the specified inclusion criteria. To focus on factors affecting fluoride release from compomer restorative materials, the search was limited to titles and abstracts using the keywords: fluoride release AND compomer. All searches followed the predefined eligibility criteria, and only full-text articles available for access were included.

### 2.5. Data Collection Process and Data Items

Five independent reviewers (M.O., A.K., W.D., J.K. and S.K.) carefully selected articles that met the inclusion criteria. The extracted data included the first author, publication year, study design, article title, compomer dental restorative materials, and the materials’ fluoride release properties. All relevant information was recorded in a standardized Excel version 15.0.4.569.1504 file.

### 2.6. Risk of Bias and Quality Assessment

In the initial stage of study selection, each reviewer independently assessed the titles and abstracts to minimize potential bias. Cohen’s k test was used to evaluate the level of agreement among reviewers. Any disagreements about including or excluding an article were resolved through discussion among the authors [34].

### 2.7. Quality Assessment

Two reviewers (J.M. and M.D.) independently evaluated the quality of the selected studies. The evaluation criteria focused on aspects such as study design, execution, and analysis, including the following details: randomization, sample size calculation, control group, detailed description of material composition, exposure time characteristics, ion release measurement method (utilization of TISAB—Total Ionic Strength Adjustment Buffer, Sigma-Aldrich, St. Louis, MO, USA), and sample storage method (environment pH/temperature—1 point; two methods—2 points). The studies were scored on a scale of 0 to 10 points, with a higher score indicating better study quality. The risk of bias was assessed as follows: 0–4 points indicated a high risk, 5–7 points indicated a moderate risk, and 8–10 points indicated a low risk. Any discrepancies in scoring were resolved through discussion until a consensus was reached.

## 3. Results

### 3.1. Study Selection

An initial database search of PubMed, Scopus, and WoS identified 287 potentially relevant articles for this review. After removing duplicates, 166 articles remained for screening. Following the initial screening of titles and abstracts, 129 articles that did not focus on fluoride release from dental compomers were excluded. Additionally, the full text of three articles was unavailable. Ultimately, 34 articles were included in the qualitative synthesis of this review. Due to the considerable heterogeneity among the included studies, conducting a meta-analysis was not feasible.

### 3.2. General Characteristics of the Included Studies

The included studies investigated fluoride release from compomer restorative materials under different experimental conditions [1,18,19,35,36,37,38,39,40,41,42,43,44,45,46,47,48,49,50,51,52,53,54,55,56,57,58,59,60,61,62,63,64,65]. To facilitate systematic comparison, the studies were grouped according to two primary methodological variables: storage conditions and pH of the storage medium.

The studies demonstrated that the storage medium plays a significant role in fluoride release. Among the analyzed research, distilled water was the most frequently used storage medium, appearing in 21 studies. Fluoride release in distilled water varied significantly, with values ranging from 0.2 ppm to 3.4 ppm, depending on the study [35,38,42]. Artificial saliva was used in nine studies [18,38,42,44,48,59,60,61,63], and comparisons showed that fluoride release was generally lower in artificial saliva than in distilled water. For instance, Sirinoglu-Capan et al. [38] reported that Dyract released 2.0 ppm of fluoride in artificial saliva (pH 7) compared to 3.4 ppm in distilled water (pH 5). The ionic composition of artificial saliva may influence fluoride retention and release. Other studies found that fluoride release was highest when stored in acidic environments, indicating the importance of pH in fluoride dynamics [19,37,59].

Another key factor affecting fluoride release was the pH of the storage solution. Several studies confirmed that fluoride release increased significantly in acidic environments (pH 4.0–5.5) [19,37,60,61]. Moreau et al. [19] demonstrated that Dyract Flow exhibited the highest cumulative fluoride release at pH 4.0, with values reaching 516 ± 6 µg/cm^2^ over 84 days. Similar findings were reported for Freedom, which released more fluoride at pH 4.3 (2.7 µg/cm^2^) than at pH 6.2 (1.2 µg/cm^2^) [60,61]. Conversely, fluoride release decreased in neutral or alkaline environments, with studies showing lower values at pH 7.0 compared to acidic conditions [18,38]. This suggests that fluoride ion diffusion from compomers is more effective in cariogenic environments where the pH is lower.

Comparisons among different compomers also revealed variability in fluoride release performance. Among commercially available materials, F-2000 exhibited the highest fluoride release, maintaining 1.62 ppm on day one and 0.11 ppm after four weeks, outperforming Dyract in long-term fluoride emission [47]. Other compomers, such as Compoglass and Glasiosite, showed varying levels of fluoride release, with Glasiosite releasing three times more fluoride than Compoglass on day one [55]. Experimental compomers tested by Adusei et al. [51] exhibited even higher fluoride release, with values exceeding 500 ppm after six weeks, though the absence of detailed storage conditions limited direct comparisons.

In addition to fluoride release, some studies examined fluoride recharge potential. Fluoride uptake from external sources, such as fluoride gels, varnishes, and toothpaste, played a role in enhancing fluoride release. Studies showed that NaF gels increased fluoride emission significantly, though this effect was temporary [55]. Senthilkumar et al. [62] found that daily brushing with fluoride toothpaste resulted in sustained fluoride release, comparable to the effect of a single fluoride varnish application. These findings highlight the potential for enhancing fluoride release through external fluoride exposure, which may be beneficial in clinical applications (Table 1).

### 3.3. Main Study Outcomes

The reviewed studies consistently indicate that fluoride release from compomer restorative materials follows a characteristic pattern. Most studies report an initial burst release, where fluoride concentrations are highest within the first 24 h, followed by a rapid decline over the next few days, and subsequent stabilization at a lower level over time [18,35,37,38,41,42]. This trend is consistent across different storage conditions, with variations observed depending on the material composition, storage medium, and pH environment.

A systematic comparison of the included studies revealed that compomers generally release less fluoride than glass ionomer cements (GICs) but more than composite resins [18,35,37,38,41,42,43,44,45,46,47,49,53,63,64]. For example, Dyract XP, Dentsply Sirona, Konstanz, Germany, one of the most extensively studied compomers, exhibited an initial fluoride release of 0.2–3.4 ppm in distilled water, with fluoride levels declining by 50–70% after 48 h [35,38,42]. By day 7, fluoride release from Dyract stabilized at approximately 0.15–0.6 ppm, which was three to five times lower than GICs but approximately twice the release rate of composite resins [18,35,37,38,41,42,44]. Similar findings were observed for other compomers, such as F-2000, which demonstrated superior fluoride release compared to Dyract XP, with 1.62 ppm recorded on day one and maintaining higher long-term fluoride release (0.11 ppm at week four compared to Dyract’s 0.06 ppm) [47].

The analysis of fluoride release trends across various compomers highlights differences in their performance. Compoglass F, Ivoclar Vivadent, Schaan, Liechtenstein, studied in five papers, showed an initial fluoride release of 10–15 µg/cm^2^/day, which declined to 1–2 µg/cm^2^/day after one week [55,56,57,58]. When exposed to sodium fluoride (NaF) gel, its fluoride release increased temporarily from 0.27 ppm to 2.4 ppm, but this effect lasted only two days [55]. On the other hand, Twinky Star, VOCO GmbH, Cuxhaven, Germany, exhibited an initial release of 0.95 ppm, which declined to 0.24 ppm by day 30 [63]. After fluoride varnish application, fluoride release increased to 1.83 ppm, significantly lower than glass ionomer sealants, which released up to 35.95 ppm [62]. Similarly, Glasiosite showed low fluoride release (0.2 ppm on day seven, which was one-fourth of Compoglass F), but after NaF treatment, its release increased to 0.37 ppm. Compared to GICs, Glasiosite released 10–15 times less fluoride, positioning it closer to composite resins than to GICs in terms of fluoride release properties [55].

The pH environment significantly influences fluoride release rates, with most compomers demonstrating increased fluoride release in acidic conditions. Freedom, for instance, released 2.7 µg/cm^2^ at pH 4.3, compared to 1.2 µg/cm^2^ at pH 6.2 [61]. This pH-dependent behavior was also observed in studies by Silva et al. [60], Sen et al. [37], and Moreau et al. [19], confirming that lower pH enhances fluoride release. Dyract Flow, tested by Moreau et al., exhibited cumulative fluoride release of 516 ± 6 µg/cm^2^ at pH 4 over 84 days, whereas fluoride levels at neutral pH were significantly lower. Conversely, studies such as those by Kosior et al. [59] and Hammouda et al. [58] confirmed that the highest fluoride release occurs within the first 24 h, after which fluoride levels stabilize for at least six months.

Beyond passive fluoride release, some studies examined the fluoride recharge potential of compomers. Daily brushing with fluoride-containing toothpaste or exposure to fluoride varnish enhanced fluoride release, albeit temporarily. Senthilkumar et al. [62] found that daily fluoride brushing resulted in sustained fluoride release comparable to a single fluoride varnish application. Similarly, Makkai et al. [63] reported that fluoride release after brushing lasted longer than after fluoride gel application. This suggests that compomers can act as fluoride reservoirs, but their recharge capacity is lower than that of GICs. Experimental compomers have also shown promise in improving fluoride release capabilities. Adusei et al. [51] tested novel compomer formulations, with one experimental compomer maintaining 527.5 ppm fluoride release after six weeks—significantly higher than conventional compomers like Dyract AP (459.2 ppm). These results suggest that modifications in compomer composition could enhance their long-term fluoride release properties, potentially bridging the gap between traditional compomers and GICs (see Table 2).

### 3.4. Quality Assessment

Of the total number of articles included in the present review, six [18,37,39,49,59,61] were classified as high quality, ranging from 8 to 9 out of 10. In contrast, twenty-three studies [1,19,35,37,38,40,41,42,43,44,45,46,48,50,51,52,54,56,57,60,63,64,65] demonstrated a moderate risk of bias, with scores falling between 5 and 7. Additionally, five studies [36,47,53,55,58] were deemed to have low quality, receiving scores ranging from 2 to 4 (refer to the Table 3).

## 4. Discussion

Fluoride release from compomers is influenced by various factors, including material composition, storage conditions, and fluoride recharge. Most studies confirm that compomers release less fluoride than glass ionomer cements (GICs) but more than composite resins [18,35,37,38,41,42,43,44,45,46,47,49,53,63,64]. However, some findings contradict this trend. Zhao et al. [57] reported no significant difference in fluoride release between compomers and composites, while Dhull et al. [65] found that giomers released more fluoride than compomers. The storage medium also affects fluoride diffusion, with some studies indicating greater release in deionized water than in artificial saliva [18,42], whereas others found the opposite [37], likely due to differences in ionic composition. A common release pattern was observed across studies, with the highest fluoride levels detected within the first 24 h, followed by a gradual decline and stabilization over several months [58,59]. Additionally, external fluoride applications—such as toothpaste, varnish, or gels—can enhance and prolong fluoride release [55,62,63]. Research on extracted teeth suggests that fluoride uptake is most effective in immature permanent teeth, particularly when pre-conditioning agents are used [48]. These findings highlight the role of compomers as materials capable of sustained fluoride release, particularly for patients at high risk of caries, where additional fluoride exposure can maximize their protective effects.

One of the most significant external factors influencing fluoride release from compomers is the pH of the surrounding environment. Studies included in this review demonstrate that fluoride release increases under acidic conditions, particularly at pH levels below 5.5, which mimic cariogenic environments [18,19,38,42,59,60,61]. Kosior et al. [59] found that compomers released the highest fluoride levels in artificial saliva at pH 4.5, whereas significantly lower levels were observed in neutral or alkaline solutions. Similarly, Şirinoğlu-Çapan et al. [38] reported enhanced fluoride release in acidic conditions, likely due to increased material degradation and ion diffusion. This trend aligns with the broader literature indicating that resin-based materials release more fluoride in acidic environments due to ion exchange and filler hydrolysis [54]. Additionally, glass ionomer-based materials, including compomers, function as pH-dependent fluoride reservoirs, releasing more fluoride during cariogenic acid attacks [66]. A study by Al-Jadwaa et al. further supported this, demonstrating that fluoride release from composite resins was significantly higher at pH 4 compared to neutral or alkaline conditions [67]. Moreover, Nigam et al. [18] observed that pH-cycling—simulating alternating periods of demineralization and remineralization—further influences fluoride release patterns, with the highest release occurring during acidic phases. While the ability of compomers to release fluoride in response to pH fluctuations enhances their cariostatic potential, maintaining a balance between fluoride release and long-term material stability remains crucial. Further research is needed to optimize formulations that ensure both durability and sustained therapeutic benefits.

Another factor influencing fluoride release from the tested compomers was the medium in which the material samples were stored. The deionized water used in many studies [1,18,35,37,38,39,40,41,42,45,46,47,52,53,54,55,56,57,58,60,61] does not accurately reflect the complex chemistry of the oral environment. Therefore, several authors also tested the materials in artificial saliva [18,37,38,42,43,44,48,59,62,63,64] that contained NaCl, KCl, urea, Na_2_S × 9H_2_O, NaH_2_PO_4_ × 2H_2_O, and calcium ions in the form of CaCl_2_ × 2H_2_O. Additionally, one study used natural saliva [49], while another utilized a 0.9% saline solution [19,59]. According to Silva et al., fluoride release from the tested restorative materials varied depending on the storage medium due to differences in ion flow mechanisms and pH levels. In most studies, the storage medium was changed daily. Kosior et al. [59] found that compomers released the highest fluoride levels in artificial saliva, while much lower levels were observed in other solutions, which was largely influenced by the pH of the medium. In contrast, Nigam et al. [18] observed that fluoride ion release was highest in the pH-cycling model across all tested restorative materials, followed by deionized water, with artificial saliva yielding the lowest release.

Temperature fluctuations in the oral cavity, caused by the consumption of hot or cold foods and beverages, make it essential to analyze fluoride release from compomer materials at different temperatures. In most studies, the tested materials were stored at 37 °C, which best represents the oral environment. However, exceptions include studies [47,53] where the temperature was 25 °C, and one study [48] where the medium was kept at room temperature. In a study by Sen et al. [18], the authors tested fluoride release at three different temperatures, 4 °C, 37 °C, and 55 °C, finding that the highest fluoride release occurred at 55 °C. In general, fluoride release increased with rising temperature, with one exception: after 14 days, fluoride release from glass ionomer cement (GIC) at 37 °C was higher than at 55 °C. Yan Z. et al. [68], in a study on glass ionomer materials, confirmed that all tested glass ionomers exhibited higher fluoride loading capacity at elevated temperatures. Additionally, increasing the ambient temperature enhanced both fluoride release and material loading capacity. These findings are crucial for developing optimized fluoride delivery strategies in restorative materials.

These findings open promising avenues for further research in this field. However, it is important to acknowledge the limitations of the current systematic review. To enhance the evaluation of this topic, further studies are warranted—preferably with larger sample sizes and including both clinical and in vivo investigations. Future research should also incorporate a broader range of commercial materials, tested over extended periods, under varying temperatures, media, and pH conditions. Additionally, it would be valuable to explore the impact of fluoride release on the antibacterial properties of these materials, as well as their physicochemical characteristics.

## 5. Conclusions

This review highlights that compomer restorative materials predictably release fluoride—starting with an initial burst, followed by a steady, lower release phase. They release more fluoride than composite resins but less than glass ionomer cement, making them a viable choice for moderate fluoride release in clinical applications. Most studies supporting these findings present a moderate risk of bias, with relatively few high-quality studies available. This gap in evidence underscores the need for more rigorous research. Their specific formulations influence fluoride release and stability in compomers. Some compomers release more fluoride in acidic conditions, while others demonstrate better recharge potential after fluoride exposure. Future studies should further explore these differences, particularly in a clinical setting, to better understand their role in cavity prevention and long-term durability.

Despite these insights, the limitations of this systematic review must be acknowledged. Additional studies are needed to provide a more comprehensive evaluation of this topic, preferably with larger sample sizes and including both clinical and in vivo investigations. Future research should also explore a broader range of commercial materials, including metal-based nanomaterials, which have emerged as promising agents for treating and preventing dental caries [69]. Evaluating these materials under varying temperatures, media, and pH conditions would enhance understanding of their clinical performance. Additionally, further studies should investigate the impact of fluoride release on these materials’ antibacterial properties and physicochemical characteristics to assess their full potential in restorative dentistry.

## Figures and Tables

**Table 1:** General characteristics of studies.

Study	Aim of the Study	Material and Methods	Results	Conclusions
Gateva V. et al. [35]	Determining which of the bioactive filling materials used in primary dentition releases the most fluoride ions	Three material samples—Fuji IX GP Extra (GIC), Dyract (compomer), and Beautifil II (giomer)—were incubated in deionized water for 24 h. Fluoride ion concentration was measured using an ionometer at 7 days and then at 2, 3, and 4 weeks.	All materials showed peak fluoride release on day one. GIC consistently released the most fluoride. Giomer released more fluoride than compomer, but not significantly.	Materials released fluoride with decreasing levels over time. GIC had highest fluoride release but suffered from weaker mechanical properties, low abrasion resistance, limited durability, and poor aesthetics. Giomers and composites performed better in these aspects.
Kosior P. et al. [59]	Comparison of the release of fluoride ions into a solution imitating the oral cavity environment by two materials—composite and compomer	The compomer Freedom and composite Wave were tested for 168 h in four environments: saline solution, deionized water, and artificial saliva with and without calcium chloride at pH levels ranging from 4.5 to 7.5. Fluoride ion release was measured using an ion-selective Orion electrode.	The Wave composite released less fluoride than the Freedom compomer. The compomer had the lowest release in saline and the highest in artificial saliva at pH 4.5, while the composite released the most in deionized water and the least in artificial saliva at pH 7.0.	Both materials released the most fluoride in an acidic environment due to surface erosion, which increased fluoride emission. The Wave composite peaked after three days, while the Freedom compomer reached its maximum on the first day.
Senthilkumar A. et al. [62]	Fluoride release from dental sealants after exposure to fluoridated toothpaste and clinically applied fluoride varnish	The study tested three dental sealants—giomer (Shofu Beautisealant), GIS (Fuji VII), and resin (Voco Twinky Star)—for fluoride release over 15 days using a fluoride ion-selective electrode. After 15 days, the first group was exposed to fluoridated toothpaste, the second to fluoride varnish, and the third was a control.	Fluoride release from resin, glass ionomer, and giomer sealants significantly increased after using fluoride toothpaste. GIS sealants released the most fluoride, followed by giomer and resin. After fluoride varnish application, GIS had the highest release, followed by giomer and resin sealants.	Fluoride ion release from all dental sealants is enhanced by daily application of fluoride toothpaste and fluoride varnish only once.
Todor L. et al. [36]	Comparison of compomers to composites and glass ionomers in the context of their clinical behavior as a dental restoration materials.	Seventy-eight patients with 228 direct restorations using compomers (Dyract AP, Dyract Extra), composite (Point 4), and glass-ionomers (Kavitan Plus, Ketac Molar) were evaluated over 3 years at five intervals using USPHS criteria, assessing caries, retention, aesthetics, integrity, and longevity.	Composites and compomers outlast glass ionomers, with better color stability and less abrasion. Compomers absorb water to offset shrinkage while releasing fluoride. Unlike glass ionomers, water does not interfere with compomer curing, improving marginal integrity.	Composites and compomers have good color matching, with Dyract Extra showing results similar to the composite. Dyract AP, with its water absorption and fluoride release, had the least secondary caries and postoperative sensitivity. Compomers improve on glass ionomers in surface texture but have limited mechanical strength in high-stress cavities. They are easy to process, bond well to tooth tissue, and have good color stability.
Sen M. et al. [37]	To evaluate and compare the effects of storage conditions, temperature, and time on the fluoride release and loading in aesthetic restorative materials.	Thirty samples of resin-modified GIC, Filtek microhybrid composite, Dyract AP compomer, and hybrid composite were tested for fluoride dynamics. Samples were stored in distilled water and artificial saliva at 4 °C, 37 °C, and 55 °C, with measurements at 1, 7, 14, and 28 days. Weekly recharging used 5 min immersion in 5000 ppm NaF solution.	The highest release was seen in temperature 55 °C in glass—ionomer, followed by compomer and composite. Fluoride release was greatest in GIC followed by Dyract AP in artificial saliva when compared to distilled water. Fluoride recharge was highest in the third week.	GIC showed the highest fluoride release and loading, followed by compomer and composites. Fluoride release was lower in distilled water than in artificial saliva and increased with temperature and time. After loading, GIC had the highest fluoride re-release, the compomer was medium, and composites had the lowest.
Şirinoğlu-Çapan B. et al. [33]	To evaluate antimicrobial activity and fluoride loading and release by materials used in pediatric dentistry for direct restorations	Fuji IX GP Capsule (GIC), Fuji II LC Capsule (resin-modified GIC), GCP Glass Fill (glass carbomer), Dyract XP (compomer), Beautifil II (giomer), and Filtek Z 250 (composite) were tested for 8-week fluoride release in artificial saliva and distilled water for fluoride recharge capability and antimicrobial activity against *S. mutans* and *L. acidophilus* using agar diffusion.	Giomer had the lowest fluoride release, followed by glass carbomer, with high-viscosity glass ionomer cement showing the highest release. Fluoride release increased significantly after reloading with APF gel and fluoride varnish. Release was higher in distilled water than artificial saliva due to the lower pH, which increased material porosity. No antimicrobial effects were observed in any of the materials.	Glass carbomer can serve as a viable alternative to glass ionomer cements for fluoride release. Fluoride release from glass ionomer materials is higher than from resin-based materials and increases as pH decreases.
Gumustas B et al. [55]	To evaluate the mechanical properties of fluoride-releasing dental materials after multiple fluoride recharges and discharges.	Thirty rectangular samples and thirty disks of Photac Fil Quick Aplicap (resin-modified glass ionomer), Ketac Molar Easymix, IonoStar Molar (glass ionomers), Compoglass F, and Glasiosite (compomers) were immersed in distilled water and divided into six groups. Fluoride release was evaluated over 24 days using ion chromatography with three 7-day discharge-recharge cycles. Flexural strength was tested at six intervals using a universal testing machine.	The microhardness and flexural strength of the tested restorative materials showed no significant changes throughout the experiment, and the testing time did not impact the flexural strength of the materials.	The mechanical properties of dental materials are not influenced by fluoride loading and release. However, fluoride ion release is greater when the material is recharged with fluoride gel. Therefore, the selection of materials for clinical use should be based on their ability to charge and release fluoride.
Wei Su L. et al. [39]	To investigate the fluoride release/loading capacity of an LDH-containing dental resin composite and its effect on physical and biological characteristics, including cytotoxicity, mechanical properties, and color changes.	The daily release of fluoride was measured over 90 days using an electrode, with fluoride loading occurring from day 30 to day 60. Materials included 3% and 5% LDH in microhybrid resin composite (wet-milled micro-LDH: R3W, R5W; dry-milled nano-LDH: R3D, R5D) with a commercial composite control. The tests, following ISO 4049, evaluated surface microhardness, cytotoxicity, Al^3+^/Li^+^ ion leaching, and color changes (L, *a*, b*).	The resin composites with LDH release and recharge fluoride more easily, with R3 and R5 showing greater stability and longevity compared to R3W, R5W, R3D, R5D, and the control. The LDH filler had no negative effect on flexural strength, and microhardness was significantly higher in R3, R5, R3D, and R5D compared to the control. The addition of LDH slightly increased the red and yellow color but did not cause cytotoxicity.	Adding the LDH filler improved the fluoride release in dental resins, with smaller LDH particles resulting in faster and greater fluoride release. LiAl-F LDH is an effective filler for dental resin composites, helping to prevent tooth decay by releasing fluoride.
Makkai Z. L. et al. [63]	To assess the effectiveness of two glass ionomers and two compomers in their ability to both release and absorb fluoride ions.	Sixty samples were prepared from Glassfill, Securafill, Glasiosite, and Twinky Star. Fifteen specimens of each material were divided into three test groups: control, fluoride varnish-treated, and dentifrice-treated. The specimens were stored in artificial saliva at 37 °C, and fluoride ion measurements were taken using an Orion fluoride meter at five time points over 30 days (days 1, 2, 3, 10, and 30).	Glassfill showed the highest fluoride release throughout the study. While the fluoride varnish caused an immediate increase within 24 h, the fluoride toothpaste showed better long-term effects, showing significant improvement after 30 days. All materials showed a pattern of high initial fluoride release, followed by a gradual decrease.	All materials release fluoride but self-curing glass ionomer cement was most effective. Fluoride varnish provided immediate but short-term effects, while fluoridated toothpaste proved more beneficial for long-term caries prevention.
Garoushi S. et al. [1]	To compare mechanical and physical properties of five commercial fluoride-releasing materials—Dyract, CompGlass, Beautifil II, ACTIVA-Restorative, and GC Fuji II LC.	The mechanical and physical properties were assessed using three-point bending tests, wear simulation, hardness measurements, and shrinkage stress analysis. Water absorption was monitored for 37 days, while fluoride release was measured over 10 days. Sample sizes ranged from two to eight specimens per test, depending on the property being evaluated.	ACTIVA-Restorative showed the highest fracture resistance, while Beautifil II showed the best flexural strength; GC Fuji II LC showed the highest fluoride release and water sorption, but the lowest flexural strength. ACTIVA-Restorative showed excellent wear resistance with minimal wear depth. Shrinkage stress was similar in all materials.	Each material has specific clinical advantages, indicating that material selection should be based on clinical requirements.
Bayrak G. D. et al. [40]	To investigate the effects of polishing methods on fluoride release, material roughness, and bacterial adhesion.	Beautifil II, GCP Glass Fill, Amalgomer CR, Dyract XP, and Fuji IX GP were tested for fluoride release, bacterial adhesion, and roughness. A total of 315 specimens was divided into control, Sof-lex, and Enhance/PoGo polishing groups. Measurements used ion-selective electrode (fluoride), profilometry (roughness), and optical density of *S. mutans* (bacterial adhesion).	Glass carbomer with Sof-lex released the most fluoride, with the highest release in the first 24 h. Glass ionomers released more fluoride than resin-based materials. Polishing increased fluoride release except in giomer. Fluoride release declined rapidly over 3 days, then stabilized. Controls had the lowest roughness (except glass carbomer), while ceramic-reinforced glass ionomer showed the highest post-polishing roughness.	Polishing significantly increased fluoride release in most restorative materials, particularly GI-based materials. No direct correlation was found between surface roughness or fluoride release and bacterial accumulation.
Tiwari S. et al. [56]	Evaluation on fluoride release and antibacterial activity of different materials.	Twenty samples of each material, made of GIC II and IX, Zirconia-Reinforced GIC, and compomer. Samples were stored in deionized water at 37 °C with fluoride ion concentration measured using a selective electrode on days 1, 7, 14, and 21. Antibacterial activity against *S. mutans* was determined using the agar diffusion test.	Zirconomer consistently demonstrated the highest fluoride release across all time intervals, while compomer showed the lowest. Fluoride release followed a pattern of initial increase by day 7, followed by a decline on days 14 and 21. Zirconomer showed the largest zone of antibacterial inhibition, while compomer showed no zone of inhibition.	Zirconomer demonstrated superior antibacterial and fluoride-releasing capabilities. It showed the largest zone of bacterial inhibition against *S. mutans* and consistently highest fluoride release across all time intervals.
Bansal R. et al. [41]	To investigate the fluoride release and recharge dynamics of different materials comparing their initial fluoride release patterns and their ability to re-release fluoride after exposure to professional and home fluoride application methods.	Sixteen specimens (fifteen per material) of GIC (Fuji II), resin-modified GIC (Fuji II LC), giomer (Beautiful II), and compomer (Dyract) were stored in deionized water. Fluoride release was measured on days 1, 7, and 15 using a fluoride electrode. Specimens were then divided into control, 2% neutral NaF, and 1000 ppm fluoride toothpaste groups, with re-release measured on days 1, 7, and 15 post-treatment.	GICs the released most fluoride, followed by resin-modified GIC, giomer, and compomer. All peaked on day one, with conventional and resin-modified GICs showing initial burst, while giomer and compomer maintained consistent release; 2% NaF recharged better than toothpaste, with conventional GIC showing best recharging ability. Re-release peaked on day one, then quickly returned to baseline.	Conventional GICs demonstrated the most effective fluoride release and recharging capability among the tested materials.
Rama Rao B. S. et al. [64]	To assess the fluoride release and uptake of five different restorative materials.	Sixty samples (twelve per material) made of GIC (Fuji II), command set (Fuji VII), resin-modified GIC (Fuji II LC), compomer (F2000), composite resin (Tetric Ceram) and divided into three subgroups: A—control; B—stored in 2% NaF 1 min; C—brushed with fluoridated dentifrice for 2 min. Fluoride release was measured using a fluoride ion-specific electrode. Measurements were taken on days 1, 7, and 28.	Both conventional and command set GICs showed similar fluoride release patterns, significantly higher than resin-modified GI, compomer, and composite resin. GICs showed fluoride absorption when exposed to NaF and fluoridated dentifrice, increasing fluoride release, while in contrast, the compomer and composite resin showed no fluoride absorption.	GICs demonstrate superior fluoride dynamics, offering continuous protection through their ability to release and recharge fluoride ions over time.
Zhao X. et al. [57]	To examine fluoride release patterns and recharge capabilities across five dental materials.	Ten samples of each material, Fuji II LC (light-cure GIC), Beautifil (giomer), Compoglass F (compomer), Charisma (composite), and an experimental resin (containing novel chelating and fluoride-releasing monomer) were stored in deionized water with daily fluoride measurements via ion-selective electrode. After 28 days, specimens were immersed in 2.0% NaF foam for 4 min, followed by daily measurements for 7 days (repeated three times).	Fuji II LC demonstrated superior fluoride release followed by experimental composite and Compoglass F. Beautifil and Charisma exhibited minimal release. After charging, all materials showed a significant increase on the first day and most returned to baseline levels within 2 days, with the exception of Fuji II LC, which maintained a higher release for 5 days.	Materials with high initial release showed better charging ability with the exception of Beautifil, which showed low initial release but good recharge ability. Charisma showed lowest recharge capacity. Experimental composite performed comparably to compomers.
Yusoff N. et al. [42]	To compare the fluoride release and fluoride accumulation from four dental restorative materials stored in deionized water and artificial saliva.	Eighty samples (twenty of each material) were prepared from Fuji VII (GIC), Fuji II LC (light-cure GIC), Dyract (compomer), and Z350 (composite) and divided into two groups—stored in deionized water and in artificial saliva—both stored at 37 °C for 5 days. Fluoride release was measured every 24 h with fluoride ion-specific electrode.	In deionized water, Fuji VII demonstrated the highest fluoride release, followed by Fuji II LC, Dyract, and Z350, maintaining this pattern consistently across 5 days. Notably lower fluoride release levels were observed in artificial saliva, but in the same ranking order. The pattern of fluoride release tended to show an initial increase followed by a gradual decrease, with the trend being more marked with deionized water.	All materials released more fluoride in deionized water than artificial saliva. Materials were ranked in terms of fluoride release from highest to lowest as follows: resin-modified GIC, conventional GIC, compomer, composite.
Gururaj M. et al. [43]	To assess the fluoride release and reuptake behavior of five dental restorative materials.	Fifty samples (ten of each material), Fuji VII (GIC), Vitremer (resin-modified GIC), Dyract (compomer), Tetric ceram (composite), and Beautifil (giomer), were stored in artificial saliva and fluoride release was measured at 6 h, 24 h, 48 h, and weekly for 5 weeks. For recharge, testing samples were exposed to 1.23% APF gel for 4 min and fluoride release was measured with fluoride ion-selective electrode at days 1, 2, 3, and 7.	GIC showed initial burst followed by decline, while resin-modified GIC maintained highest sustained release. Compomer, giomer, and composite displayed moderate release with delayed peaks. GIC and resin-modified GIC demonstrated superior recharge ability, with fluoride composite showing minimal recharge. Both GIC types performed best overall, while compomer and giomer showed comparable but lower performance.	All tested materials demonstrated fluoride release and recharge capabilities, with resin-modified GIC showing the highest consistent performance, while composite showed the lowest.
Gjorgievska E. et al. [44]	To determine the extent to which ions released from fluoride-containing dental restorations migrate through the enamel and dentine of extracted teeth.	Forty extracted human third molars were randomly divided into four groups and restored with Fuji IX, Fuji II LC, Dyract AP, and Unifil Flow (eight samples each in 32 mm molds). SEM and EDX assessed elemental distribution (Na, Al, Sr, F, Mg, Si, P, and Ca) at 1 month and 18 months.	Fuji IX showed highest elemental incorporation (Si, Sr, and F in enamel/dentine at 1 month; Mg, Na, and F in dentine at 18 months). Fuji II LC showed increased Na in enamel/dentine and Mg/P in dentine after 18 months. Dyract AP showed Na/Mg in enamel and F in dentine after 18 months. Unifil Flow showed minimal migration (only Na/Mg in enamel; no F in dentine). Fuji IX demonstrated most extensive incorporation overall.	Fluoride-releasing materials cause ion migration into adjacent tissues, with conventional GIC showing most extensive migration (F, Al, Si, and Na). Resin-modified GIC shows less migration, while compomers and fluoridated composites have minimal migration, with fluoridated composites failing to promote fluoride uptake. Effective caries inhibition requires ion uptake for clinical benefit.
Hammouda I. M. et al. [58]	The impact of water storage on fluoride release and the mechanical properties of a compomer restorative material.	Composan Glass material (PROMEDICA, Neumünster, Germany) was used to prepare five samples (5 × 2 mm), which were cured with a halogen light-curing device (Spring Power, IL, USA). Fluoride release was measured at intervals of 24 h, 1 week, 1 month, 2 months, 3 months, and 6 months using a fluoride-specific electrode. The bending properties and fracture strength of the samples were evaluated with a universal testing machine, while Vickers hardness was assessed using a microhardness tester.	Fluoride release significantly decreased from 24 h to 6 months. Flexural strength increased slightly for 1 week, then declined. Fracture toughness improved initially, then decreased over time. Vickers hardness increased until 1 month, then dropped by 6 months. Fluoride release at 6 months significantly affected flexural strength, flexural modulus, and Vickers hardness.	Compomer shows an initial rapid increase in fluoride release during the first week, which then decreases to a level of release that remains relatively stable for 6 months.
Neelakantan P. et al. [45]	To evaluate the fluoride release of a novel glass ionomer-based nano-ionomer compared to other restorative materials and to assess the impact of nano-sized fillers on fluoride release.	A total of 60 samples (n = 10 per group) were prepared from conventional GIC, resin-modified GIC (RMGIC), nano-ionomer, compomer, fluoride-releasing composite, and a control composite. Light-cured materials were cured with an Elipar 2500 halogen lamp for 20 s (RMGIC, nano-ionomer, and composite) or 40 s (compomer) on both sides. After 24 h at 37 °C, 95% humidity, samples were immersed in 5 mL buffered deionized water (pH 7) at 37 °C. Fluoride release was measured with a selective ion electrode daily for one week, then at days 14, 21, and 28, using TISAB III buffer.	GIC showed the highest fluoride release in the first three days, followed by Nano, RMGIC, and CP, with RC having the lowest. GIC released significantly more fluoride than others, with no significant difference between RMGIC and Nano. RC showed no detectable release. GIC, RMGIC, and Nano showed sharp drops after week one, stabilizing by weeks three/four, while CP and RC maintained steady low levels. Cumulative release was highest for GIC, RMGIC, and Nano. GIC and RMGIC showed no significant differences in the first two weeks, but all groups differed significantly by weeks three/four.	GIC showed the highest fluoride release, followed by Nano and RMGIC, with a sharp decline after the first week. CP and RC had the lowest release, with RC showing none. Overall, GIC, RMGIC, and Nano released significantly more fluoride than CP and RC.
Moreau J. L. et al. [19]	To examine how solution pH and duration of immersion influence the mechanical properties and fluoride release of restorative materials.	Three resin-modified glass ionomers (Viremer, Fuji II LC, and Ketac Nano), one compomer (Dyract Flow), and one composite (Heliomolar), were tested. Flexural strength and elastic modulus were measured before and after 84 d of immersion in solutions of pH 4, 5.5, and 7. The release of F was evaluated in relation to pH levels and duration of immersion.	Immersion duration and material type significantly affected mechanical properties. Vitremer’s flexural strength dropped from 99 ± 25 MPa to 32 ± 9 MPa after 84 days, while Heliomolar showed a smaller decrease (99 ± 9 MPa to 65 ± 7 MPa). Fuji II LC’s strength remained stable across pH levels, but fluoride release increased significantly at lower pH, reaching 609 ± 25 µg/cm^2^ at pH 4 compared to 188 ± 9 µg/cm^2^ at pH 7.	The restoratives examined in the study demonstrated the capacity to significantly enhance the release of fluoride (F) at acidic, cariogenic pH levels. The mechanical properties of these F-releasing restoratives underwent substantial degradation when subjected to immersion testing.
Mousavinasab S. M. et al. [46]	To ascertain the quantities of fluoride emanating from materials containing fluoride. Four glass ionomer cements (Fuji IX, Fuji VII, Fuji IX Extra, and Fuji II LC), a compomer (Dyract Extra), and a giomer (Beautifil) were utilized.	Twenty cylindrical specimens were prepared from each material. The amount of fluoride released was measured during the first week and on days 14 and 21. This was achieved by using a specific fluoride electrode and an ion analyzer. Statistical analysis was conducted using analysis of variance (two-way ANOVA) and Tukey–Kramer multiple comparison tests (*p* = 0.05).	A statistically significant variation was observed in the fluoride release of different materials over time (*p* < 0.05). The maximum cumulative fluoride release of days 1–7 was observed to be related to Fuji VII, followed by Fuji IX Extra, Fuji II LC, Fuji IX, Dyract Extra, and Beautifil in descending order. This order remained unchanged until the 21st day.	Higher amounts of fluoride were released from Fuji IX, Fuji VII, Fuji IX Extra, and Fuji II LC compared to Beautiful and Dyract Extra. The study suggests that the extent of the glass ionomer matrix plays an important role in determining the fluoride-releasing ability of glass ionomer cement materials.
Dhull KS et al. [65]	To ascertain the extent of fluoride release from both giomer and compomer when subjected to varying topical fluoride regimens. Furthermore, the study seeks to make a comparison between the amount of fluoride released from giomer and compomer.	Forty-eight giomer and compomer specimens were divided into four groups: control, fluoridated dentifrice (500 ppm) once daily, twice daily, and dentifrice plus fluoridated mouthwash (225 ppm) once daily. Each specimen was immersed in a demineralizing solution for 6 h, followed by an 18 h remineralization phase. Fluoride release was measured daily in both solutions for 7 days, with total daily release calculated by summing the amounts released in both phases.	Giomer releases more fluoride (ppm) compared to compomer. Both giomer and compomer release significantly greater amounts of fluoride in an acidic demineralizing solution than in a neutral remineralizing solution. Increased fluoride exposure significantly enhances fluoride release from both giomer and compomer.	The order of fluoride release from different exposure regimens was as follows: fluoridated dentifrice twice daily > fluoridated dentifrice once daily + fluoridated mouthwash > fluoridated dentifrice once daily > control group. Giomer showed comparatively greater fluoride recharge/uptake capabilities than compomer.
Nigam A. G. et al. [18]	To examine the pattern of fluoride release from glass ionomer cement, resin-modified glass ionomer cement, compomer, and composite resin when stored in various storage media.	Sixty samples, with fifteen samples each for four different materials, were prepared, and five samples of each material were suspended in 4 mL of four different solutions: deionized water, artificial saliva, and two pH-cycling solutions (demineralizing solution with pH 4.3 and remineralizing solution with pH 7.0). The fluoride ion release was measured over a 15-day period, with readings taken on days 1, 2, 5, 9, and 15. Fluoride concentrations in the solutions were measured using an Orion fluoride ion-specific electrode.	Substantial variation was identified in the quantity of fluoride released from the various materials under different storage conditions. Glass ionomer cement exhibited a significantly higher level of fluoride release across all storage media. A highly significant difference was also observed between composite resin and the other materials, with composite resin demonstrating a markedly lower level of fluoride release in all media.	ART glass ionomer cement exhibited the highest fluoride release across all media types, followed by resin-modified glass ionomer cement, compomer, and composite resin. The pH-cycling model was the most effective in enhancing fluoride release, with deionized water and artificial saliva showing lower release levels. These results highlight the significant impact of the surrounding pH on fluoride release from dental materials.
Gjorgievska E. et al. [48]	To assess how aluminum and fluoride release differ between different fillings placed in different tooth types and how tooth type affects the overall pattern of ion release.	Class V cavities were made in primary and permanent teeth. They were divided into groups and filled with Fuji IX, Fuji II LC, Dyract AP, and Unifil Flow with or without conditioning. The teeth were stored in artificial saliva for a month and then their ion levels were tested using spectrophotometry.	The differences between conditioned and unconditioned teeth were significant only for Dyract AP. The aluminum/fluoride release coefficient was highest for glass ionomer cements and lowest for composite resin. For each material, it was higher for primary than permanent teeth.	The interaction with ions released by restorative materials depends on the type of tooth.
Al-Naimi O. T. et al. [49]	To assess whether fluoride release under different conditions can inhibit biofilm formation.	Samples of Unifil-S, Reactmer, Dyract AP, Fuji II LC, and Ketac-Fil Plus Aplicap were prepared and placed in natural saliva at pH 7.1 or 3.8. Daily fluoride release in saliva was measured using an ion-selective electrode.	During the first two and a half weeks, the resin-modified glass ionomer cement released more fluoride than the composite and compomer combined, but this difference decreased over time.	In both acidic and neutral environments, GICs released higher amounts of fluoride than composite, giomer, and compomer.
Al-Sakarna B. K. et al. [47]	Measurement of fluoride released from glass ionomer cements and compomers in vitro using a fluoride ion-detecting electrode.	Samples of Ketac-Fil, Fuji IX GP, Fuji VII, Fuji II LC, Vitremer, Dyract AP, and F-2000 were prepared. They were placed in deionized water and fluoride concentration was applied for 3 months using an ion-specific fluoride electrode.	Both compomers released less fluoride than glass ionomers. Dyract AP showed the lowest fluoride release per day of all materials.	Compomers released less fluoride than glass ionomer cements. All compomers released similar amounts of fluoride, with F 2000 showing the highest and Dyract AP the lowest emission.
Silva K. G. et al. [60]	Evaluation of surface microhardness and fluoride release from restorative materials stored in distilled/deionized water or pH-cycling.	Samples were prepared from Ketac-Fil Plus, Vitremer, Fuji II LC, Freedom, and Fluorofil and subjected to pH-cycling or stored in distilled water for 15 days. The amount of fluoride released was measured using a fluoride-specific electrode.	Ketac-Fil Plus and Fluorofil released more fluoride in water, while Vitremer, Fuji II LC, and Freedom did so at an average pH of 4.6.	Fluoride release from the tested materials varies depending on the storage medium.
Silva K. G et al. [61]	Evaluation of the effect of demineralizing solutions with different pH on fluoride release and the properties and hardness of the surface of restorative materials.	Samples were prepared from Ketac-Fil Plus, Vitremer, Fuji II LC, Freedom, and Fluorofil, then subjected to pH-cycling (4.3; 4.6; 5.0; 5.5; and 6.2) and immersed in demineralizing and remineralizing solutions for 15 days. Fluoride was measured with a fluoride-specific electrode.	At pH 4.3 and 4.6, Vitremer showed the highest fluoride release, followed by Ketac-Fil Plus, Fuji II LC, Fluorofil, and Freedom.	Changing the pH of the demineralization solution affects the release of fluoride from the tested materials.
Marczuk-Kolada G. et al. [50]	Evaluation of fluoride ion release and antibacterial activity in two groups of restorative dental materials.	Fuji IX and Dyract AP samples were prepared and placed in 10 mL of pH 6.8 phosphate buffer. Fluoride ion concentrations were measured after 1, 4, 7, 14, 30, and 60 days using direct potentiometry with an Orion fluoride ion-selective electrode.	Both materials showed a cumulative increase in fluoride ion concentration in solutions with a peak on day 7. Fuji IX achieved higher concentration.	Both tested materials release fluoride ions, which influences their antibacterial activity.
Adusei G. O. et al. [51]	Determination of the properties of two compomers formulated with vinylphosphonic acid (VPA) monomer.	Experimental formulations of compomers (A and B) were obtained from a standard formulation of composite resin for dental applications with the addition of VPA monomer. Dyract AP was used for comparison. Samples were stored in 5 mL of liquid for up to 6 weeks. Fluoride concentration was determined in the presence of a buffer to adjust the total ionic strength using a fluoride-selective electrode.	Compomer A released the highest amounts of fluoride, followed by Dyract AP and compomer B.	VPA has the potential to be used as an acid functional monomer in compomers.
Kavaloglu Cildir S. et al. [52]	To investigate the fluoride release and re-accumulation behavior of conventional glass ionomer cements and polyacid-modified composite resins after contact with mouthwash and toothpaste.	Samples were made of the following materials: Ionofil Plus, Fuji IX, Dyract AP, and Hytac Aplitip; they were then stored in distilled water. Fluoride measurements were made using a fluoride ion-selective electrode after the 1st, 2nd, 3rd, 4th, 7th, 14th, 21st, and 28th days.	During the study, Ionofil Plus released the most fluoride, followed by Fuji IX, and Hytac Aplitip released the least amount.	Compomers did not show an initially high level of fluoride ion release but maintained a low and relatively constant level.
Yldz M et al. [53]	To determine the fluoride release from glass ionomer cements and compomer materials after 1, 7, 30, and 120 days of storage in deionized water.	Samples made of Hytac, Dyract, Aqua Ionofil, and Ceramfil β materials were stored in distilled water. Fluoride concentration was measured after 1st, 7th, 30th, and 120th days by the colorimetric alizarin method.	From day 7 to day 30, fluoride release was higher for glass ionomer cements. From day 30 to day 120, it was higher for compomers. Ceramfil β had the highest fluoride release overall.	The initial and sustained fluoride release rate of the Dyract compomer was lower than that of the two glass ionomer cements and another compomer, Hytac, confirming the results of studies by other authors.
Itota T et al. [54]	To investigate the ability of resin-based materials containing fluorinated glass filler to recharge and release fluoride.	Samples prepared with Reactmer paste, Dyract AP, and Xeno CF were stored in distilled water for 38 days. Fluoride ion concentration was measured using a calibrated fluoride-specific electrode.	The cumulative amounts of total and free fluoride ions released from the materials in decreasing order were as follows: Reactmer paste > Dyract AP > Xeno CF.	The extent of the glass ionomer matrix of the glass filler played an important role in the ability of the resin-based materials to release and recharge fluoride.

**Table 2:** Detailed characteristics of studies.

Authors	Material (Type/Producer)	Storage Conditions/Solutions pH	Fluoride Measurement Method	Fluoride Release Timeline	Fluoride Release Results	Effect of pH on Fluoride Release
Gateva V. et al. [35]	Dyract XP, (Dyract XP, Dentsply Sirona, Konstanz, Germany)	5 mL of deionized water in temperature 37 °C ± 0.5 °C.	Ion-selective electrode for fluorides connected to ion meter PerfectIONTM	24 h, every day for a week and then on the 14th, 21st, and 28th days	Compomer values were lower compared to GICs. Dyract, mean (ppm) Day 1 = 0.6 Day 2 =0.54 Day 3 = 0.39 Day 4 = 0.29 Day 5 = 0.2 Day 6 = 0.16 Day 7 = 0.15 Day 14 = 0.1 Day 21 = 0.08 Day 28 = 0.07	No data
Kosior P. et al. [59]	Freedom (F) (SDI, Hamilton Parkway Itasca, Australia)	-Saline solution -Artificial saliva solution with the addition of CaCl_2_ × 2H_2_O in the pH range of 4.5 and 5.5 -Artificial saliva free of calcium chloride in the pH range of 4.5, 5.5, 6.0, 7.0, and 7.5 -Deionized water -All solutions were 37 °C	Ion-selective Orion model 9609 electrode +microcomputer pH/ion meter CPI-551 Elmetron	7 days = 168 h -Measurements were taken after 3, 24, 48, 72, 69, and 168 h	Freedom (F) -Highest cumulative release of fluoride ions into artificial saliva solution at pH 4.5 (31.195 ± 10.461 μg F/mm^2^) -Lowest into saline solution (3.694 ± 1.115 μg F/mm^2^) -The levels of fluoride released from the Freedom (F) composite material were considerably higher in comparison to those released from the Wave (W) composite material	Freedom reached its maximum fluoride release on the first day in the acidic environment of pH 4.5–5.5
Senthilkumar A. et al. [62]	Twinky star (VOCO GmbH, Cuxhaven, Germany)	Synthetic saliva (pH 5.3), 37 °C.	Fluoride ion-selective electrode	Total duration—30 days Measurement every 24 h during first 15th days Recharging: Group A—toothpaste 458 ppm 2xday Group B—750 ppm once Group C—control Monitored at 30 days	After 15 days: TS—1.45 ppm Giomer—27.82 GIS—42.81 Recharging: Group A: TS—1.83 ppm Giomer—35.95 GIS—33.74 Group B: TS—1.25 ppm Giomer—28.57 GIS—39.63 Group C: TS—1.09 ppm Giomer—25.98 GIS—31.73	No data
Todor L. et al. [36]	Dyract AP, Dyract Extra (Dentsply Sirona, Konstanz, Germany)	In vivo, 78 patients with 228 direct restorations.	The USPHS criteria	Tested after 3, 6, 12, 24, and 36 months	Dyract AP, with its water absorption triggering acid–base reactions, showed the least damage from secondary caries and postoperative sensitivity after curing	No data
Sen M. et al. [37]	Dyract AP (Dentsply Sirona, Konstanz, Germany)	Distilled water and artificial saliva at 4 °C, 37 °C, and 55 °C.	Fluoride-sensitive electrode pH/Ion 510, Oakton	Total duration: 28 days 4 measurements taken on Day 1, 7, 14, and 28 Fluoride recharge day—1, 8, and 15	The highest release was seen on day 14, temperature 55 °C—7.083 ppm Results in 4 °C: Day 1—0.077 DW, 0.136 AS Day 7—0.158 DW, 0.406 AS Day 14—0.196 DW, 1.927 AS Day 28—0.302 DW, 0.384 AS Results in 37 °C: Day 1—0.772 DW, 0.302 AS Day 7—1.008 DW, 1.092AS Day 14—1.173 DW, 3.990 AS Day 28—1.588 DW, 1.310 AS Results in 55 °C: Day 1—0.650 DW, 0.667 AS Day 7—3.003 DW, 1.827 AS Day 14—3.026 DW, 7.083 AS Day 28—7.328 DW, 2.880 AS Fluoride rerelease after recharging in AS, day 7 in third week—6.50 ppm vs DW 1.04	Higher fluoride release in artificial saliva (pH of 5.3 to 5.5) than distilled water—37 °C, day 14—3.990 ppm vs 1.173 ppm
Şirinoğlu-Çapan B. et al. [38]	Dyract XP (Dentsply Sirona, Konstanz, Germany)	Artificial saliva (pH = 7.0) and distilled water (pH = 5.0) Temperature: 37 °C	Fluoride-specific electrode Orion 9690BN	Total duration—8 weeks Measurements—after 2 h; 1, 2, and 7 days; then weekly for 7 weeks Recharging—after 8 weeks Measurements—after 2 h; 1, 2, and 7 days; then weekly for 7 weeks	All tested materials (except for composite) show the highest fluoride release rates on the first day, which then fell on the second day; then, on the seventh day, the release rates increased again Dyract XP: 1 day ~3.4 ppm DW, ~2.0 ppm AS 2 day~ 1.5 ppm DW, ~0.5 ppm AS 7 day~2.5 ppm DW, ~2.0 ppm AS 56 day ~1.0 ppm DW, ~1.0 ppm AS After recharging Dyract XP control group 2 h ~0.2 ppm DW, ~0.2 ppm AS 2 day ~0.2 ppm DW, ~0.2 ppm AS 7 day ~1.0 ppm DW, ~1.0 ppm AS After recharging Dyract XP APF gel 2 h ~1.8 ppm DW, ~1.25 ppm AS 2 day ~0.5 ppm DW, ~0.5 ppm AS 7 day ~1.1 ppm DW, ~1.2 ppm AS After recharging Dyract XP fluoride varnish group 2 h ~3.0 ppm DW, ~2.0 ppmAS 2 day ~2.0 ppm DW, ~0.2 ppm AS 7 day ~1.8 ppm DW, ~1.8 ppm AS	DW (pH = 5.0) AS (pH = 7.0)
Gumustas B et al. [55]	Compoglass F (VOCO GmbH, Cuxhaven, Germany) Glasiosite (VOCO GmbH, Cuxhaven, Germany)	5 mL deionized water changed daily at 37 °C Recharging materials were taken for 4 min in a neutral sodium fluoride gel 2% (9000 ppm).	Ion chromatography (IC) (DX 100, Dionex, Camberley, UK-IC)	Total duration—24 days 6 measurements taken on day 7 (T1), 8 (T2), 15 (T3), 16 (T4), 23 (T5), and 24 (T6) Tested before and 1 day after recharging at day 7, 15, and 23	Fluoride Release—Compoglass mean ± SD (median) T1 0.27 ± 0.07 bB (0.24) T2 2.4 ± 0.55 cA (2.4) T3 0.6 ± 0.17 aB (0.65) T4 1.98 ± 0.64 bA (2.23) T5 0.33 ± 0.08 bB (0.38) T6 2.29 ± 0.33 aA (2.27) Fluoride release—Glasiosite mean ± SD (median) T1 0.2 ± 0.33 bA (0.06) T2 0.33 ± 0.22 eA (0.22) T3 0.2 ± 0.15 cA (0.12) T4 0.3 ± 0.23 cA (0.3) T5 0.18 ± 0.09 aA (0.2) T6 0.37 ± 0.06 cA (0.39)	No data
Wei Su L. et al. [39]	Dyract flow (Dentsply Sirona, Konstanz, Germany)	3 mL deionized water at 37 °C.	Ion-selective electrode (Orion 9609 BNWP, Thermo Fisher Scientific, Waltham, MA, USA)	Tested every day over 90 days (daily fluoride recharging from 30th to 60th day)	Compomer showed rapid fluoride release initially, then decreased to levels similar to microhybrid flowable resin composite (0.85 ppm) Fluoride release rose from 0.03 to 0.14 ppm during days 31–60, then declined rapidly with no recharge by days 61–90	No data
Makkai Z. L. et al. [63]	Glasiosite (VOCO GmbH, Cuxhaven, Germany) Twinky Star (VOCO GmbH, Cuxhaven, Germany)	1 mL artificial saliva per specimen (pH = 7, 37 °C), the storage medium changed daily.	Orion 720A fluoride meter with 9609 BN fluoride ion electrode 1 mL artificial saliva mixed with 1 mL TISAB II buffer solution Calibration: standard solutions between 0.1 ppm and 10 ppm fluoride	Total duration: 30 days 5 measurements taken on day 1, 2, 3, 10, and 30	Compomer group values were lower compared to GICs Glasiosite samples released more fluoride than Twinky Star in control groups Twinky Star -Control group: day 1: 0.95 ppm to day 30: 0.24 ppm -Varnish-treated group: peaked at day 2 with 129.75 ppm (*p* = 0.0002, significantly higher than other materials) -Dentifrice-treated group: day 1: 0.65 ppm to day 30: 0.94 ppm Glasiosite -Control group: day 1: 1.06 ppm to day 30: 0.61 ppm -Varnish-treated group: day 2 spike to 48.22 ppm -Dentifrice-treated group: day 1: 1.39 ppm to day 30: 0.87 ppm	No data
Garoushi S. et al. [1]	Dyract (Dentsply Sirona, Konstanz, Germany) CompGlass (Ivoclar Vivadent, Schaan, Liechtenstein)	5 mL deionized water per specimen (37 °C); storage medium changed daily.	Orion ion analyzer with ion-specific electrode (Orion Research, Boston, MA, USA) 5 mL storage water mixed with 0.5 mL TISAB III buffer solution Calibration: fluoride standards ranging from 0.2 to 100 ppm	Total duration: 10 days 10 measurements: every 24 h for 10 days Fresh deionized water used after each measurement	Both compomers -Initial release (Dyract: ~4 ppm CompGlass: ~4.5 ppm) was lower than GICs (~12–13 ppm) with no burst effect -Maintained more constant release levels compared to glass ionomers -Days 3–7: ~1–2 ppm -Day 10: ~0.5–1 ppm	No data
Bayrak G. D. et al. [40]	Dyract XP (Dentsply Sirona, Konstanz, Germany)	3 mL deionized water per specimen, (37 ± 0.5 °C); storage medium changed daily.	Ion-selective electrode (ISE) (Orion Ionplus, 96-09BN) 0.4 mL TISAB III buffer added at 10 vol% to provide background ionic strength Calibration: sodium fluoride standard solutions (10 ppm, 1 ppm, 0.1 ppm)	Total duration: 28 days 7 measurements taken on days 1, 2, 3, 7, 14, 21, and 28	Dyract XP (compomer): -Day 1: 1.9 ± 0.2 ppm (control), 2.3 ± 0.4 ppm (Sof-lex), 2.9 ± 0.4 ppm (Enhance/PoGo) -Day 2: 0.6 ± 0.08 ppm (control), 0.7 ± 0.1 ppm (both polishing systems) -Day 28: 0.1 ± 0.02 ppm (control), 0.3 ± 0.1 ppm (Sof-lex), 0.5 ± 0.1 ppm (Enhance/PoGo) Significantly lower than GICs (*p* < 0.05) No significant difference between polishing systems on day 2 (*p* = 0.168), but significant by day 28 (*p* < 0.0001) Release was much lower than GICs, with no burst effect (GCP Glass Fill: 28.7 ± 3.3 ppm on day 1)	No data
Tiwari S. et al. [56]	Compoglass (Ivoclar Vivadent, Schaan, Liechtenstein)	1 mL deionized water per specimen (37 °C); storage medium changed daily.	Fluoride ion-selective electrode meter (Orion 940, Expotech, Texas, USA) 1:1 dilution with TISAB III buffer Calibration: using standard fluoride solutions (0.20, 1.00, 2.00, 10.00, 20.00, and 100 ppm)	Total duration: 21 days 4 measurements taken on days 1, 7, 14, and 21	Compoglass: -Day 1: 2.11 ± 0.01 ppm -Day 7: 3.13 ± 0.01 ppm -Day 14: 2.00 ± 0.02 ppm -Day 21: 1.07 ± 0.04 ppm Showed significantly lower fluoride release compared to other materials (*p* < 0.001) Consistently showed lowest fluoride release among all materials tested at all time points	No data
Bansal R. et al. [41]	Dyract (Dentsply Sirona, Konstanz, Germany)	Specimens stored in 20 mL deionized water (37 °C); storage medium changed daily.	Ion-selective electrode (ISE) attached to an ion meter TISAB solution controls pH and prevents fluoride complexes Analysis made using ANOVA and T HSD (Tukey’s multiple post hoc procedure)	Initial release measured for 15 days; 3 measurements taken on days 1, 7, and 15 After recharging, measured for another 15 days; 3 measurements taken on days 1, 7, and 15	Dyract 1. Initial release: -Day 1: ~0.4 ppm -Day 7: ~0.3 ppm -Day 15: ~0.2 ppm 2. After recharging -Day 1: ~0.7 ppm (toothpaste), ~1.0 ppm (2%NaF) -Day 7: ~0.4 ppm (toothpaste), ~0.5 ppm (2%NaF) -Day 15: ~0.2 ppm (toothpaste), ~0.3 ppm (2%NaF) Compomer had lower recharging ability (*p* < 0.05) The professional regime (2% NaF) outperformed the home regime (toothpaste) (*p* < 0.05)	No data
Rama Rao B. S. et al. [64]	F2000 (3M ESPE, St. Paul, MN, USA)	Specimens stored in 25 mL artificial saliva (37 °C, pH = 7); storage medium changed daily Artificial saliva composition: 1 mM calcium chloride, 1 mM sodium dihydrogen phosphate, 35 mM sodium chloride, 15 mM sodium acetate.	Fluoride ion electrode (9609 BN Orion Research) coupled to microprocessor ion analyzer (EA 940 Orion Analyzer, Orion Research, Inc., Boston, USA) 10 mL saliva mixed with 10% TISAB buffer Calibration: standard solutions between 0.1 ppm and 10 ppm fluoride	Total duration: 28 days 3 measurements taken on days 1, 7, and 28	Control group: -Day 1: 49.47 μg/cm^2^ -Day 7: 5.13 μg/cm^2^ -Day 28: 4.88 μg/cm^2^ After NaF treatment: -Day 1: 49.18 μg/cm^2^ -Day 7: 16.77 μg/cm^2^ -Day 28: 10.57 μg/cm^2^ Significantly lower fluoride release compared to glass ionomers (*p* < 0.05)	No data
Zhao X. et al. [57]	Compoglass F (Ivoclar Vivadent, Schaan, Liechtenstein)	Specimens stored in 5 mL deionized water (37 °C); storage medium changed daily.	Compound fluoride ion-selective electrode (model 209, Mettler Toledo Co) and SevenMulti™ conductivity meter 5 mL TISAB II buffer added to provide constant background ionic strength Calibration: standard fluoride solutions (0.010, 0.100, 1.00, 10.0, and 100 ppm F)	Initial release: 28 days with daily measurements After recharge: 7 days of measurements Recharge process repeated 3 times	Initial release: -Day 1–7: 10–15 μg/cm^2^/day -Days 8–28: Gradual decline to 1–2 μg/cm^2^/day After recharge with 2% NaF: -Day 1 post-recharge: sharp increase to ~20 μg/cm^2^/day -Days 2–7: rapid decline to baseline levels Statistical significance: significantly lower than glass ionomer (*p* < 0.01), significantly higher than composite resin (*p* < 0.01)	No data
Yusoff N. et al. [42]	Dyract (Dentsply Sirona, Konstanz, Germany)	Samples stored in 6 mL deionized water or 6 mL artificial saliva (37 °C), sample size: 10 discs per group; storage medium changed daily.	Fluoride-specific ion electrode (ISE meter) TISAB used to decomplex contaminating ions Calibration: standard fluoride solutions (0.1, 1.0, and 10 ppm)	Total duration: 5 days 5 measurements taken every 24 h	In deionized water: -Day 1: 0.20 ppm (IQR 0.21) -Day 2: 0.33 ppm (IQR 0.15) -Day 3: 0.37 ppm (IQR 0.14) -Day 4: 0.45 ppm (IQR 0.19) -Day 5: 0.47 ppm (IQR 0.19) In artificial saliva: -Day 1: 0.04 ppm (IQR 0.00) -Day 2: 0.05 ppm (IQR 0.02) -Day 3: 0.05 ppm (IQR 0.02) -Day 4: 0.05 ppm (IQR 0.02) -Day 5: 0.06 ppm (IQR 0.00) Significant difference in fluoride release between deionized water and artificial saliva (*p* < 0.001)	No data
Gururaj M. et al. [43]	Dyract (Dentsply Sirona, Konstanz, Germany)	5 mL artificial saliva (wet mouth, ICPA) per specimen Sample size: 10 disk specimens	Fluoride ion-specific electrode (ORION, 94098N), Orion microprocessor ion analyzer (Orion Research, Inc., Boston, USA) TISAB used to stabilize pH Calibration: standard fluoride solutions (0.1, 0.5, 1, 5, and 10 ppm)	Initial measurements at 6 h, 24 h, and 48 h Then weekly intervals for 5 weeks After recharge: measurements on 1st, 2nd, 3rd, and 7th day	Initial release: -6 h: 0.33 ppm -24 h: 0.67 ppm -48 h: 0.78 ppm -1st week: 0.92 ppm -2nd week: 0.73 ppm -3rd week: 0.64 ppm -4th week: 0.65 ppm -5th week: 0.61 ppm After recharge with APF gel: -Day 1: 0.87 ppm -Day 2: 0.72 ppm -Day 3: 0.64 ppm -Day 7: 0.67 ppm ANOVA and Tukey HSD test showed statistically significant differences between all materials	No data
Gjorgievska E. et al. [44]	Dyract AP (Dentsply Sirona, Konstanz, Germany)	Artificial saliva	SEM-BEI/EDX (scanning electron microscopybackscattered electron image/energy dispersive analysis with X-rays)	Initial measurement at 1 month Last measurement at 18 months	Ion migration after 1 month from Dyract AP compomer: a. Enamel incorporation of fluorine (F.57) b. Dentine fluorine (F.69) Ion migration after 18 months of application of Dyract AP compomer: a. Enamel incorporation of fluorine (F.178) b. Dentine fluorine (F.217)	No data
Hammouda I. M. et al. [58]	Composan Glass (Ivoclar Vivadent, Schaan, Liechtenstein)	The specimens were stored in 4 mL deionized water at 37 °C.	An ion-specific electrode (Orion Electrode, Orion Research Inc., Boston, MA, USA) Calibration from 0.1 to 100 ppm Each 4 mL storage water and the 1 mL used for washing were mixed with 4 mL (TISAB) solution	24 h 1 week 1 month 2 months 3 months 6 months	24 h—2.33 ppm 1 w—2.28 ppm 1 month—1.86 ppm 2 months—1.73 ppm 3 months—1.47 ppm 6 months—1.03 ppm A decrease being noted between 24 h and 1, 2, 3, and 6 months	No data
Neelakantan P. et al. [45]	Dyract F (Dentsply Sirona, Konstanz, Germany)	The materials were stored at 95% humidity and 37 °C for 24 h to set. Each specimen was immersed in 5 mL buffered deionized water (pH 7) at 37 °C, with TISAB III added.	A combination fluoride ion-selective electrode (Model 96-09-BN, Orion Research Inc, Boston, MA, USA) connected to an Orion ion analyzer (Model EA 940, Orion Research, Inc., Boston, USA)	Measured every 24 h for the first seven days, and on days 14, 21, and 28 Total duration: 28 days	Mean fluoride release (μg/dL/mm^2^) during the first week: 4.2 ± 0.21—day 1 2.1 ± 0.04—day 2 2.5 ± 0.10—day 3 2.4 ± 0.19—day 4 1.8 ± 0.08—day 5 1.5 ± 0.09—day 6 1.5 ± 0.08—day 7 Fluoride release was highest on days 1 and 2 Compomer released fluoride at a low but constant rate throughout the study	No data
Moreau J. L. et al. [19]	Dyract Flow (Dentsply Sirona, Konstanz, Germany)	A 133 mmol/L NaCl solution was buffered to pH 4, 5.5, and 7 with 50 mmol/L lactic acid, acetic acid, and HEPES, respectively. Three specimens were immersed in 50 mL at a 2.9 mm^3^/mL ratio.	F concentration was measured with a F ion-selective electrode (Orion, Cambridge, MA)	1 day (d) 2 d 3 d 7 d and then after each 7 days Total duration: 84 days	At 84 days and pH 4, cumulative F release for Dyract Flow was 516 ± 6 g/cm^2^ Initial release was high (97 ± 8 g/cm^2^/day at 1 day), followed by a lower long-term rate (0.34 ± 0.28 g/cm^2^/day)	F release peaked early, then slowed, highest at pH 4 By 70–84 days, rates were similar across all pHs for Dyract Flow
Mousavinasab S. M. et al. [46]	Dyract Extra (Dentsply Sirona, Konstanz, Germany)	The specimens were incubated in a 95% relative humidity environment at 37 °C for 24 h. Then, they were immersed in 1 mL deionized water in the incubator at 37 °C.	A fluoride ion-selective electrode (Ion Check 45, Radiometer Analytical, Lyon, France)	1 day (d) 2 d 3 d 4 d 5 d 6 d 7 d 14 d 21 d The procedure was repeated daily, measuring cumulative fluoride release in weeks 1, 2, and 3	Cumulative fluoride release from tested materials (μg/cm^2^); standard deviations are given in parenthesis. D1 = 1.42 (0.41) D2 = 2.07 (0.53) D3 = 2.74 (0.58) D4 = 3.20 (0.58) D5 = 3.60 (0.55) D6 = 4.20 (0.59) D7 = 4.60 (0.56) D14 = 7.55 (0.69) D21= 9.62 (0.75)	No data
Dhull KS et al. [65]	No data = generic compomer	In deionized water in airtight plastic containers, at 37 °C, for three days. A demineralizing solution (CaCl_2_x2H_2_O—2.2 mM; NaH_2_PO_4_x2H_2_O—2.2 mM; and CH_3_COOH—0.05 M; pH adjusted with 1 M KOH to pH 4.4) A remineralizing solution (CaCl_2_x2H_2_O—1 mM; NaH_2_PO_4_x2H_2_O—1 mM; NaCl—35 mM; CH_3_COONax3H_2_O—15 mM; pH adjusted with 1 M KOH to pH 7 Each specimen was stored in 10 mL demineralizing solution at 37 °C for 6 h, then in 10 mL remineralizing solution at 37 °C for 18 h.	The Sartorius Professional Meter PP 25 with a “Combination” ISE (ion-selective electrode)	Total duration: 7 days Measurement every day	Daily fluoride release (ppm) from compomer Compomer only D1 = 6.74 D2 = 5.10 D3 = 2.63 D4 = 2.59 D5 = 2.03 D6 = 1.90 D7 = 1.82 Compomer + fluoridated dentifrice once daily D1 = 6.95 D2 = 5.41 D3 = 3.01 D4 = 3.05 D5 = 2.50 D6 = 2.17 D7 = 2.04 Compomer + fluoridated dentifrice twice daily D1 = 7.39 D2 = 6.06 D3 = 3.74 D4 = 3.68 D5 = 2.99 D6 = 2.70 D7 = 2.64 Compomer + fluoridated dentifrice once daily + fluoridated mouthwash D1 = 7.15 D2 = 5.76 D3 = 3.41 D4 = 3.25 D5 = 2.73 D6 = 2.32 D7 = 2.22	The mean fluoride release for compomer was significantly greater when immersed in the demineralizing solution (pH 4.4) than in the remineralizing solution (pH 7.0)
Nigam A. G. et al. [18]	Dyract (Flow) (Dentsply Sirona, Konstanz, Germany)	The studied media were deionized water, artificial saliva, and solutions for pH-cycling (demineralizing solution pH 4.3 and remineralizing solution pH 7.0), at 4 mL of their respective media.Temperature incubation: 37 ± 0.5 °C.	Orion digital ion analyzer, model (1260), equipped with combination Orion fluoride ion-specific electrode (96-09) After calibrating the electrode with 1 and 10 ppm fluoride standards, fluoride release was estimated from 5 mL samples mixed with 0.5 mL TISAB-III	Measurement points: 1 day (d) 2 d 5 d 9 d 15 d Total duration: 15 days	Deionized water (μg F/cm^2^): D1 (day 1) = 21.938 + 1.069 D2 = 10.65 + 2.178 D5 = 6.894 + 1.056 D9 = 2.456 + 0.381 D15 = 2.214 + 0.432 Artificial saliva (μg F/cm^2^): D1 (day 1) = 9.99 + 1.129 D2 = 5.378 + 0.988 D5 = 2.464 + 0.539 D9 = 1.258 + 0.225 D15 = 1.054 + 0.169 Demineralizing solution (μg F/cm^2^) pH 4.3: D1 (day 1) = 39.226 + 1.086 D2 = 29.09 + 0.481 D5 = 33.188 + 0.661 D9 = 30.434 + 0.323 D15 = 39.552 + 1.098 Fluoride release peaked on day 1, highest in the pH-cycling model (demineralizing solution)	No data
Gjorgievska E. et al. [48]	Dyract AP (Dentsply Sirona, Konstanz, Germany)	Artificial saliva at room temperature.	Spectrophotometry after isolation of the fluorides with distillation	After 1 month	Deciduous 10.22 (0.80) ppm Deciduous (conditioned) 11.28 (1.14) ppm Young permanent 13.13 (0.78) ppm Young permanent (conditioned) 13.36 (0.69) ppm	No data
Al-Naimi O. T. et al. [49]	Dyract AP (Dentsply Sirona, Konstanz, Germany)	Natural saliva at pH 7.1 or 3.8, 37 °C.	Fluoride ion-selective electrode (model 720A, Orion Research Inc. Beverly, MA, USA)	Every day for 4 weeks	Cumulative fluoride release pH 7.1 = 10.1 (0.5) ppm pH 3.8 = 211.0 (15.5) ppm	More fluoride ions were released at pH 3.8 than 7.1
Al-Sakarna B. K. et al. [47]	Dyract AP (Dentsply Sirona, Konstanz, Germany) F-2000 (3M ESPE, St. Paul, MN, USA)	Deionized water, 25 °C.	Ion-specific fluoride electrode (Orion 920A Ion analyzer)	After 24 h, weekly for the rest of the first month, and monthly for the remaining three months	Dyract AP 24 h = 0.47 (0.16) Week 1 = 0.07 (0.02) Week 2 = 0.06 (0.01) Week 3 = 0.07 (0.01) Week 4 = 0.06 (0.01) Month 2 = 0.07 (0.04) Month 3 = 0.05 (0.01) Month 4 = 0.06 (0.01) F-2000 24 h = 1.62 (0.86) Week 1 = 0.3 (0.19) Week 2 = 0.18 (0.14) Week 3 = 0.19 (0.13) Week 4 = 0.11 (0.06) Month 2 = 0.1 (0.05) Month 3 = 0.08 (0.05) Month 4 = 0.07 (0.05)	No data
Silva K. G. et al. [60]	Freedom (SDI Limited, Bayswater, Victoria, Australia) Fluorofil (Bisco, Inc. Schaumburg, IL, USA).	Distilled water or for 6 h in demineralizing solution (2.0 mmol/L Ca and P, in acetate buffer 75 mmol L-1, pH 4.6) and 18 h in remineralizing solution (Ca 1.5 mmol L-1, P 0.9 mmol/L, KCl 150 mmol/L in Tris buffer 20 mmol L/L, pH 7.0).	Fluoride-specific electrode (Orion 9609-BN; Orion Research, Inc., Beverly, MA, USA) and digital ion analyzer (Orion 720 A; Orion Research, Inc.)	Every 6 and 18 h for 15 days	Mean at distilled water: Fluorofil = 4.4 (1.3) μg F/cm^2^ Freedom = 1.2 (1.5) μg F/cm^2^ Mean at pH 4.6: Fluorofil = 3 (3.4) μg F/cm^2^ Freedom = 2.1 (1.7) μg F/cm^2^	Freedom releases more fluoride in pH 4.6 Fluorofil releases more fluoride in neutral pH
Silva K. G et al. [61]	Freedom (SDI Limited, Bayswater, Victoria, Australia) Fluorofil (Bisco, Inc. Schaumburg, IL, USA).	6 h in demineralizing solution (2.0 mmol/L Ca and P, in acetate buffer 75 mmol/L, pH 4.3; 4.6; 5.0; 5.5 or 6.2) and 18 h in remineralizing solution (Ca 1.5 mmol/L, P 0.9 mmol/L, KCl 150 mmol L-1 in Tris buffer 20 mmol/L, pH 7.0). Temp. 37 °C.	Fluoride-specific electrode (Orion 9609-BN; Orion Research, Inc., Beverly, MA, USA) and digital ion analyzer (Orion 720 A; Orion Research, Inc.)	Every day for 15 days	Mean fluoride release: Freedom pH 4.3 = 2.7 (2.1) μg F/cm^2^ pH 4.6 = 2.1 (1.7) μg F/cm^2^ pH 5 = 1.9 (1.5) μg F/cm^2^ pH 5.5 = 1.5 (1.4) μg F/cm^2^ pH 6.2 = 1.2 (1.4) μg F/cm^2^ Fluorofil pH 4.3 = 6 (4.3) μg F/cm^2^ pH 4.6 = 3 (3.4) μg F/cm^2^ pH 5 = 1 (1.3) μg F/cm^2^ pH 5.5 = 0.6 (0.4) μg F/cm^2^ pH 6.2 = 0.6 (0.3) μg F/cm^2^	Both materials release the most fluoride in pH 4.3
Marczuk-Kolada G. et al. [50]	Dyract AP (Dentsply Sirona, Konstanz, Germany)	pH 6.8 phosphate buffer, 37 °C.	Direct potentiometry with an Orion fluoride ion-selective electrode	After 1, 4, 7, 14, 30, and 60 days	No numeric values	No data
Adusei G. O. et al. [51]	Dyract AP (Dentsply Sirona, Konstanz, Germany) Experimental compomer A Experimental compomer B	Liquid—not specified.	Fluoride-selective electrode (Type PSE, EDT Instruments, Ohio, USA)	After 1 day, 1 week, 2 weeks, 4 weeks, and 6 weeks	Dyract AP 1 day = 314.7 ppm 1 week = 378.5 ppm 2 weeks = 389.3 ppm 4 weeks = 441.5 ppm 6 weeks = 459.2 ppm Compomer A 1 day = 314.7 ppm 1 week = 416.9 ppm 2 weeks = 477.7 ppm 4 weeks = 510.5 ppm 6 weeks = 527.5 ppm Compomer B 1 day = 265.4 ppm 1 week = 333.1 ppm 2 weeks = 344 ppm 4 weeks = 354 ppm 6 weeks = 370.6 ppm	No data
Kavaloglu Cildir S. et al. [52]	Dyract AP (Dentsply Sirona, Konstanz, Germany) Hytac Aplitip (3M ESPE, Minnesota, USA)	Distilled water, 37 °C.	Orion Research fluoride-specific electrode (No. 9609BN) connected to an ion analyzer (Fisher Scientific Accumet 950 pH/ion meter)	After 1st, 2nd, 3rd, 4th, 7th, 14th, 21st, and 28th days	Mean fluoride release Dyract AP Day 1 = 2.91 (0.62) Day 2 = 0.95 (0.23) Day 3 = 0.66 (0.12) Day 4 = 0.53 (0.11) Day 7 = 0.46 (0.13) Day 14 = 0.69 (0.27) Day 21 = 0.28 (0.16) Day 28 = 0.19 (0.09) Hytac Aplitip Day 1 = 1.87 (0.5) Day 2 = 0.32 (0.06) Day 3 = 0.19 (0.03) Day 4 = 0.15 (0.02) Day 7 = 0.13 (0.03) Day 14 = 0.13 (0.04) Day 21 = 0.062 (0.03) Day 28 = 0.05 (0.03)	No data
Yldz M et al. [53]	Dyract AP (Dentsply Sirona, Konstanz, Germany) Hytac Aplitip (3M ESPE, Minnesota, USA)	Distilled water, 25 °C.	The colorimetric alizarin method, spectrophotometer (Shimadzu 160 Å, Kyoto, Japan)	After 1st, 7th, 30th, and 120th days	No numeric values	No data
Itota T et al. [54]	Dyract AP (Dentsply Sirona, Konstanz, Germany)	Distilled water, 37 °C.	Fluoride-specific electrode (Combination Electrode 96-09BN, Orion Research) attached to an ion meter (model 720A, Orion Research) Ion chromatography (DX 100, Dionex, Camberley, UK)	38 days	Cumulative fluoride release Total fluoride ions = 11.66 (2.04) µg/cm^2^ Free fluoride ions = 9.54 (1.05) µg/cm^2^	No data

**Table 3:** Quality assessment of included studies.

Authors	RANDOMIZATION	Sample Size Calculation	Control Group	Detailed Description of Material Composition	Exposure Time Characteristics	Ion Release Measurement Method:	The Utilization of TISAB (Total Ionic Strength Adjustment Buffer)	Sample Storage Method:	Total Points	Risk of Bias
						No Data—0 Point		No Data—0 Point		
						Ion-Selective Electrode/SEDEDX/ Spectrophotometry/ Chromatography —1 Point		Environment pH/Temperature—1 Point		
						2 Methods—2 Points		2 Methods—2 Points		
Kosior P. et al. [59]	0	1	1	1	1	2	0	2	8	Low
Sen M. et al. [37]	1	1	1	1	1	1	1	2	9	Low
Wei Su L. et al. [39]	0	0	1	1	1	2	1	2	8	Low
Nigam A. G. et al. [18]	1	0	1	1	1	1	1	2	8	Low
Al-Naimi O. T. et al. [49]	1	0	1	1	1	2	1	2	9	Low
Silva K. G et al. [61]	1	0		1	0	2	1	2	8	Low
Gateva V. et al. [35]	0	0	1	1	1	1	1	1	6	Moderate
Senthilkumar A. et al. [62]	0	0	0	1	1	1	1	2	6	Moderate
Şirinoğlu-Çapan B. et al. [38]	0	0	1	1	1	1	1	2	7	Moderate
Makkai Z. L. et al. [63]	0	0	1	1	1	1	1	2	7	Moderate
Garoushi S. et al. [1]	0	0	1	1	1	2	1	1	7	Moderate
Bayrak G. D. et al. [40]	1	0	1	1	1	1	1	1	7	Moderate
Tiwari S. et al. [56]	0	0	1	0	1	1	1	1	5	Moderate
Bansal R. et al. [41]	0	0	1	0	1	1	1	1	5	Moderate
Rama Rao B. S. et al. [64]	0	0	1	0	1	1	1	1	5	Moderate
Zhao X. et al. [57]	0	0	1	1	1	1	1	0	5	Moderate
Yusoff N. et al. [42]	0	0	1	1	1	1	1	1	6	Moderate
Gururaj M. et al. [43]	0	0	1	1	1	1	1	0	5	Moderate
Gjorgievska E. et al. [44]	1	0	1	1	1	1	0	0	5	Moderate
Neelakantan P. et al. [45]	0	0	1	1	1	1	1	2	7	Moderate
Moreau J. L. et al. [19]	0	0	1	1	1	1	0	2	6	Moderate
Mousavinasab S. M. et al. [46]	0	0	1	0	1	1	1	1	5	Moderate
Dhull KS et al. [65]	0	0	1	0	1	1	1	2	6	Moderate
Gjorgievska E. et al. [48]	1	0	1	1	0	1	0	1	5	Moderate
Silva K. G. et al. [60]	1	0	1	0	1	1	1	2	7	Moderate
Marczuk-Kolada G. et al. [50]	0	0	1	1	1	1	1	2	7	Moderate
Adusei G. O. et al. [51]	0	0	1	1	1	1	1	1	6	Moderate
Kavaloglu Cildir S. et al. [52]	1	0	1	1	1	1	1	1	7	Moderate
Itota T et al. [54]	0	0	1	1	1	2	1	1	7	Moderate
Todor L. et al. [36]	0	0	1	1	0	0	0	0	2	High
Gumustas B et al. [55]	0	0	0	1	1	1	0	1	4	High
Hammouda I. M. et al. [58]	0	0	0	0	1	1	1	1	4	High
Al-Sakarna B. K. et al. [47]	0	0	1	0	1	1	0	1	4	High
Yldz M et al. [53]	0	0	1	0	1	0	0	1	3	High

## Data Availability

Not applicable.
